# Supplementary material for: Lactylation of METTL16 promotes cuproptosis via m6A-modification on FDX1 mRNA in gastric cancer
Source: Nat Commun. 2023 Oct 20;14:6523. doi: 10.1038/s41467-023-42025-8 (PMC10589265; doi:10.1038/s41467-023-42025-8)
Supplement: Supplementary file 1 — Supplementary Information [file 41467_2023_42025_MOESM1_ESM.pdf]

Supplementary Information

Lactylation of METTL16 promotes cuproptosis via m^{6}A-modification on *FDX1* mRNA in gastric cancer

Lianhui Sun, [1,2,7] Yuan Zhang,[1,7] Boyu Yang,[3,7] Sijun Sun,[1,7] Pengshan Zhang,[1] Zai Luo,[1] Tingting Feng,[4] Zelin Cui,[5] Ting Zhu,[2] Yuming Li,[6] Zhengjun Qiu,[1] Guangjian Fan,[2,*] and Chen Huang[1,*]

Supplementary Figures 1-7

Supplementary Fig. 1. Pathological correlation of highly concentrated copper in GC.

Supplementary Fig. 2. METTL16 is a critical mediator of cuproptosis.

Supplementary Fig. 3. METTL16 promotes cuproptosis by targeting FDX1 via m^{6}A modification.

Supplementary Fig. 4. LC-MS/MS analysis showed the lactylated and acetylated sites on METTL16 protein sequence.

Supplementary Fig. 5. Potential impact of K229 lactylation on METTL16.

Supplementary Fig. 6. SIRT2-mediated METTL16 deacetylation has no effect on the methyltransferase activity of METTL16.

Supplementary Fig. 7. AARS1/2 directly regulate the lactylation modification of METTL16.

Supplementary Data 1-2

Data 1. The changed genes at m^{6}A levels or mRNA levels after METTL16-knockdown.

Data 2. The METTL16 binding proteins were detected by the LC-MS/MS.

Supplementary Figure 1

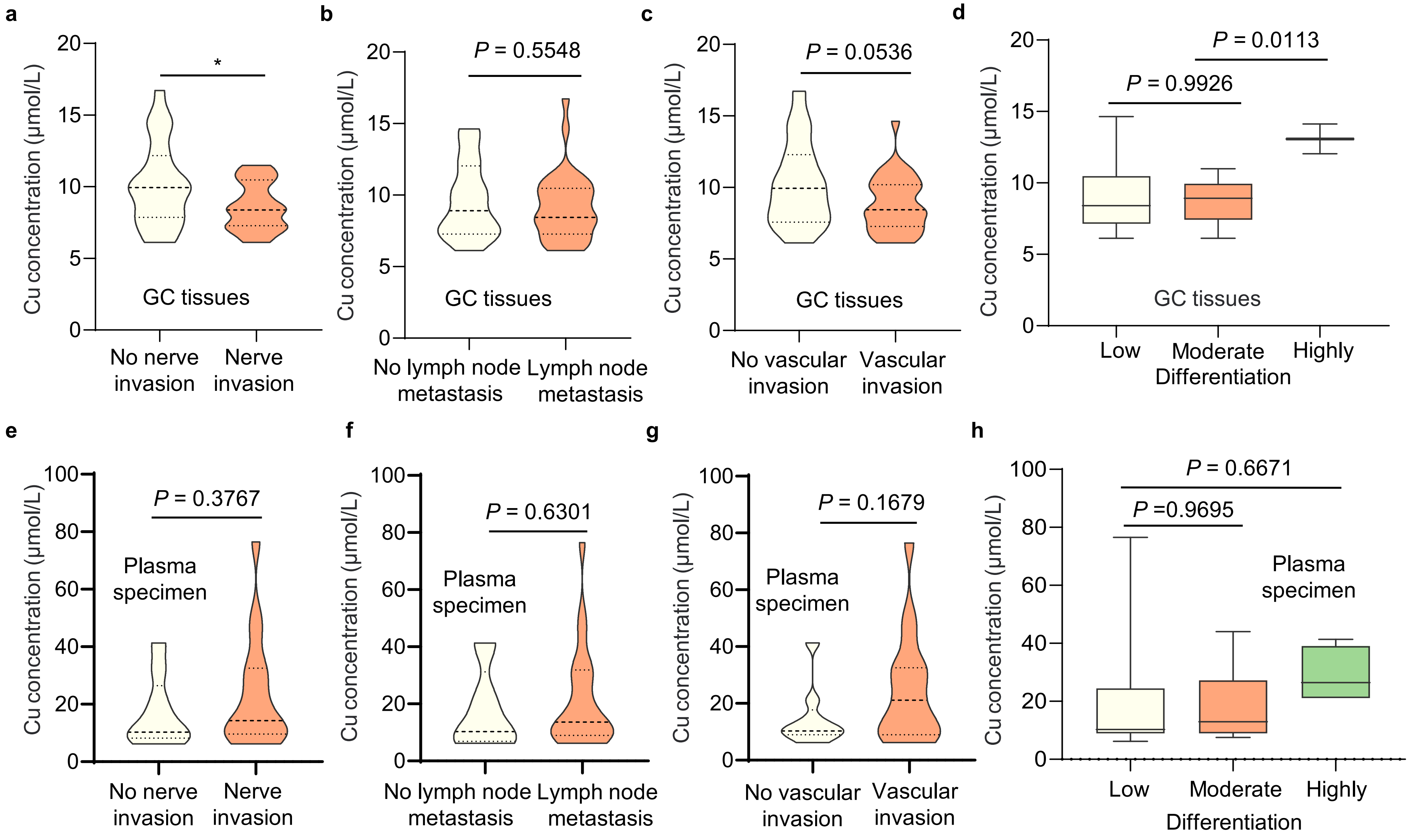

Supplementary Fig. 1. Pathological correlation of highly concentrated copper in GC. (a-d) The correlations were measured in 48 pairs of GC and adjacent normal tissue samples. **(a)** Correlation between Cu concentrations and nerve invasion (P < 0.05). **(b)** Correlation between Cu concentrations and lymph node metastasis (P = 0.5548). **(c)** Correlation between Cu concentrations and vascular invasion (P = 0.0536). **(d)** Correlation between Cu concentration and differentiation. **(e-h)** Correlations measured in GC plasma samples. **(e)** Correlation between Cu concentration and nerve invasion (P = 0.3767). **(f)** Correlation between Cu concentration and lymph node metastasis (P = 0.6301). **(g)** Correlation between Cu concentration and vascular invasion (P = 0.1679). **(h)** Correlation between Cu concentration and tumor differentiation. Statistical data presented in this figure show mean values ± SD of three times of independent experiments. Statistical significance was determined by Two-tailed t test, *P < 0.05, **P < 0.01. Source data are provided as a Source data file.

Supplementary Figure 2

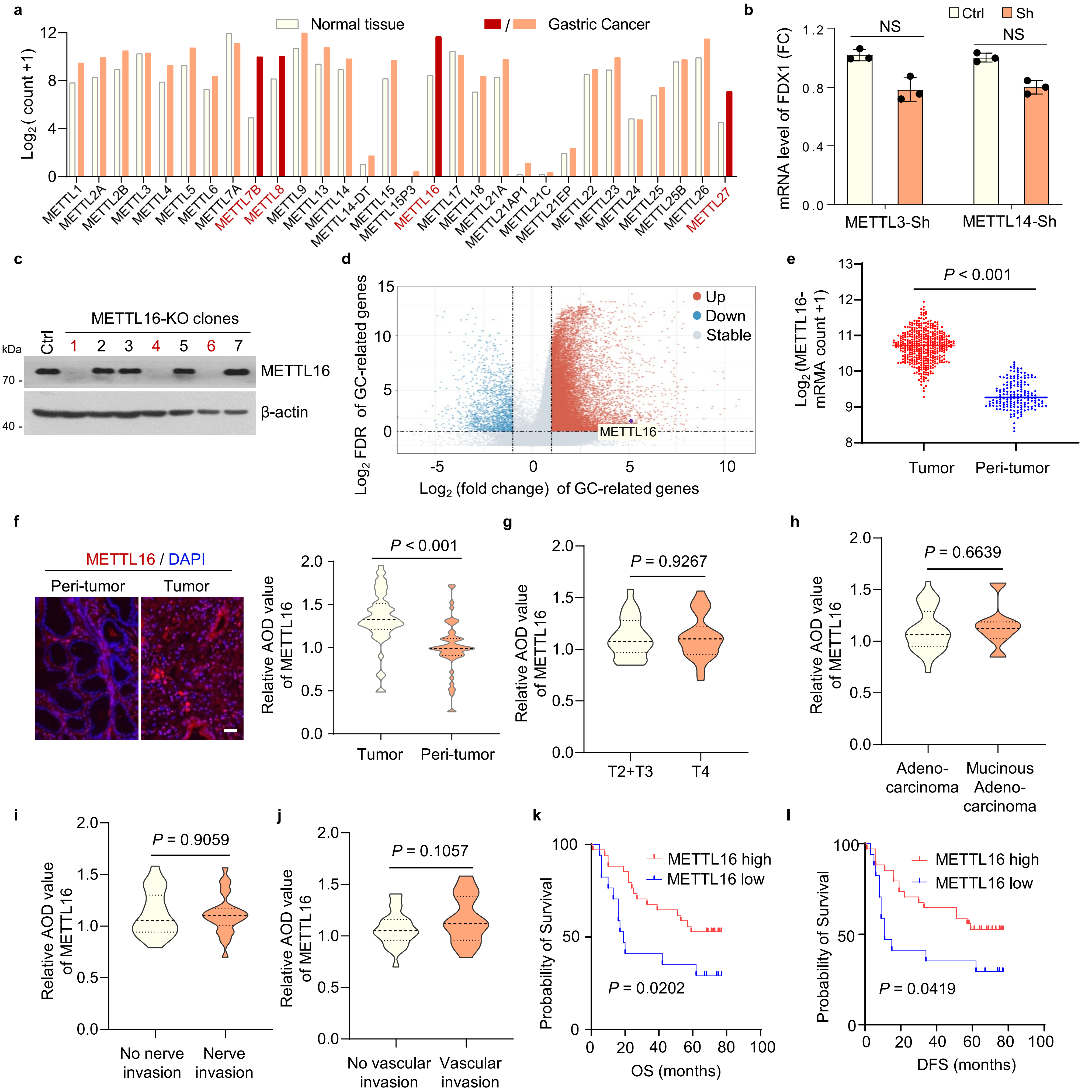

Supplementary Fig. 2. METTL16 is a critical mediator of cuproptosis. **(a)** Expression of METTL16 among members of the METTL family. Datasets were downloaded and analyzed from the TCGA and GTEx databases. **(b)** qRT-PCR analysis of *FDX1* mRNA levels in METTL3 and METTL14 knockdown cells (n=3). $P = 0.06531$ and 0.05288. **(c)** Western blot showing the knock-out efficiency of seven METTL16-KO clones. **(d-e)** Analysis of METTL16 expression using datasets from the TCGA and GTEx database. **(d)** Volcano plot showing significantly upregulated and downregulated genes in patients with gastric cancer. **(e)** Scatter plot showing *METTL16* mRNA expression in tumor and peri-tumor tissues. $P < 0.0001$. **(f)** Immunofluorescent staining of METTL16 in the tissue microarray containing 54 pairs of GC and adjacent normal tissues (left). The average optical density (AOD) values of METTL16 in tumor and peri-tumor tissues were shown on the right. $P < 0.0001$. **(g-j)** Correlation between METTL16 and pathological characteristics. Scale bar, 50 μm. **(g)** Correlation between METTL16 expression with T staging in GC. **(h)** METTL16 expression in mucinous and non-mucinous adenocarcinomas. **(i)** Correlation between nerve invasion and METTL16 expression in GC. **(j)** Correlation between vascular invasion and METTL16 expression in GC. **(k, l)** Survival analysis of Overall Survival (OS) and Disease-free Survival (DFS) in GC patients. **(k)** Difference in Overall Survival (OS) between patients with high and low METTL16 expression (HR = 2.158). **(l)** Difference in Disease-free Survival (DFS) between patients with high and low METTL16 expression (HR = 2.107). Statistical data presented in this figure show mean values ± SD of three times of independent experiments. Statistical significance was determined by Two-tailed t test, $^{*}P < 0.05$, $^{**}P < 0.01$, NS = not significant. Source data are provided as a Source data file.

Supplementary Figure 3

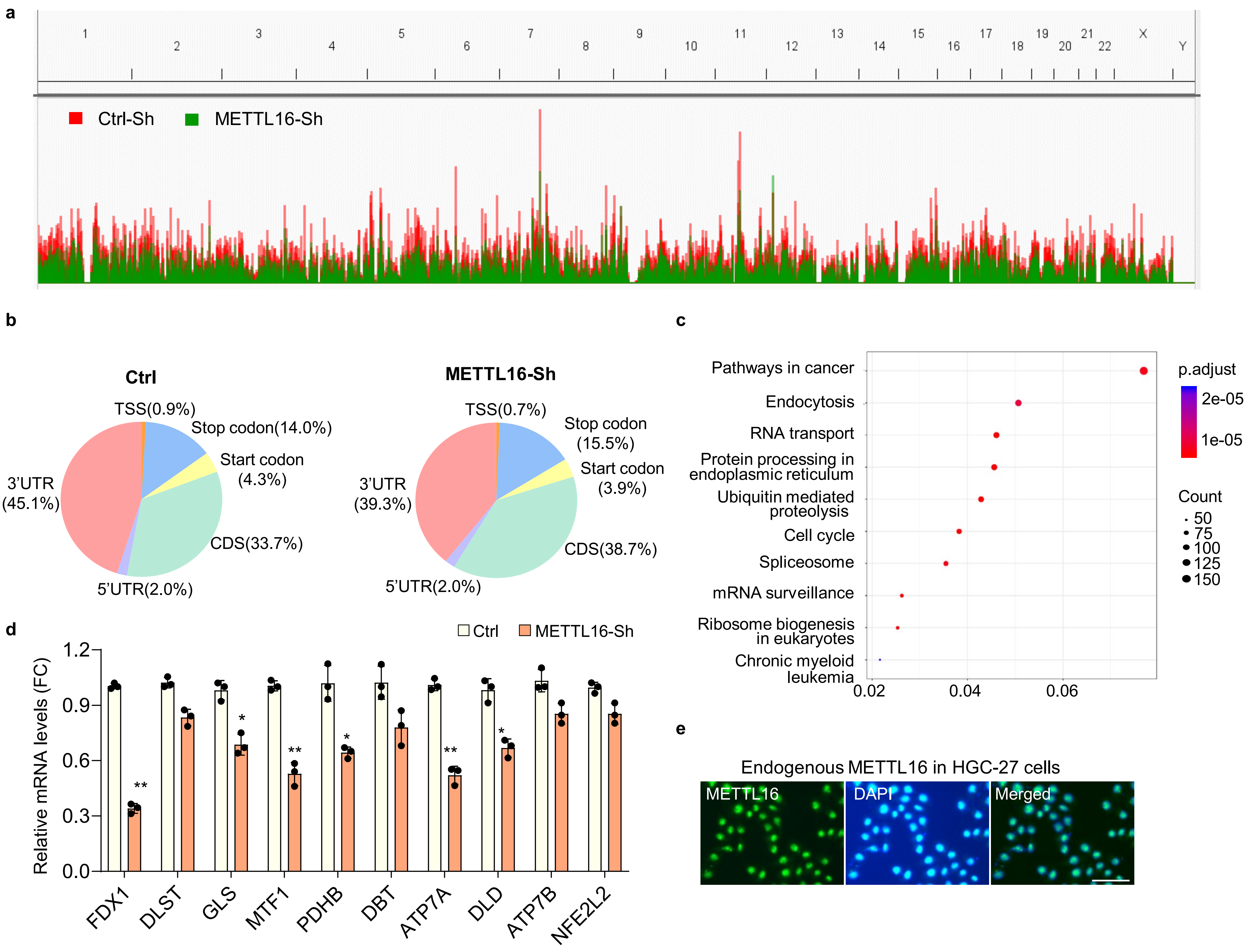

Supplementary Fig. 3. METTL16 promotes cuproptosis by targeting FDX1 via m^{6}A modification. **(a)** Metagene plot showing the distribution of m^{6}A peaks on mRNA in Ctrl and METTL16-Sh cells. **(b)** Pie plot showing the proportions of m^{6}A distribution in TSS, 3'UTR, 5'UTR, CDS, start codon and stop codon regions of mRNA in Ctrl and METTL16-Sh samples. **(c)** KEGG analysis of significantly enriched pathways in Ctrl and METTL16-Sh samples. **(d)** qRT-PCR analysis of mRNA levels of cuproptosis-related genes in Ctrl-Sh and METTL16-Sh cells (n=3). *P* (from left to right): 0.0008, 0.05351, 0.01014, 0.00435, 0.0168, 0.13572, 0.00385, 0.03083 and 0.05095. **(e)** Immunofluorescence staining of endogenous METTL16 in HGC-27 cells. Scale bar, 50 μm. Statistical data presented in this figure show mean values ± SD of three times of independent experiments. Statistical significance was determined by Two-tailed t test, *P < 0.05, **P < 0.01. Source data are provided as a Source data file.

Supplementary Figure 4

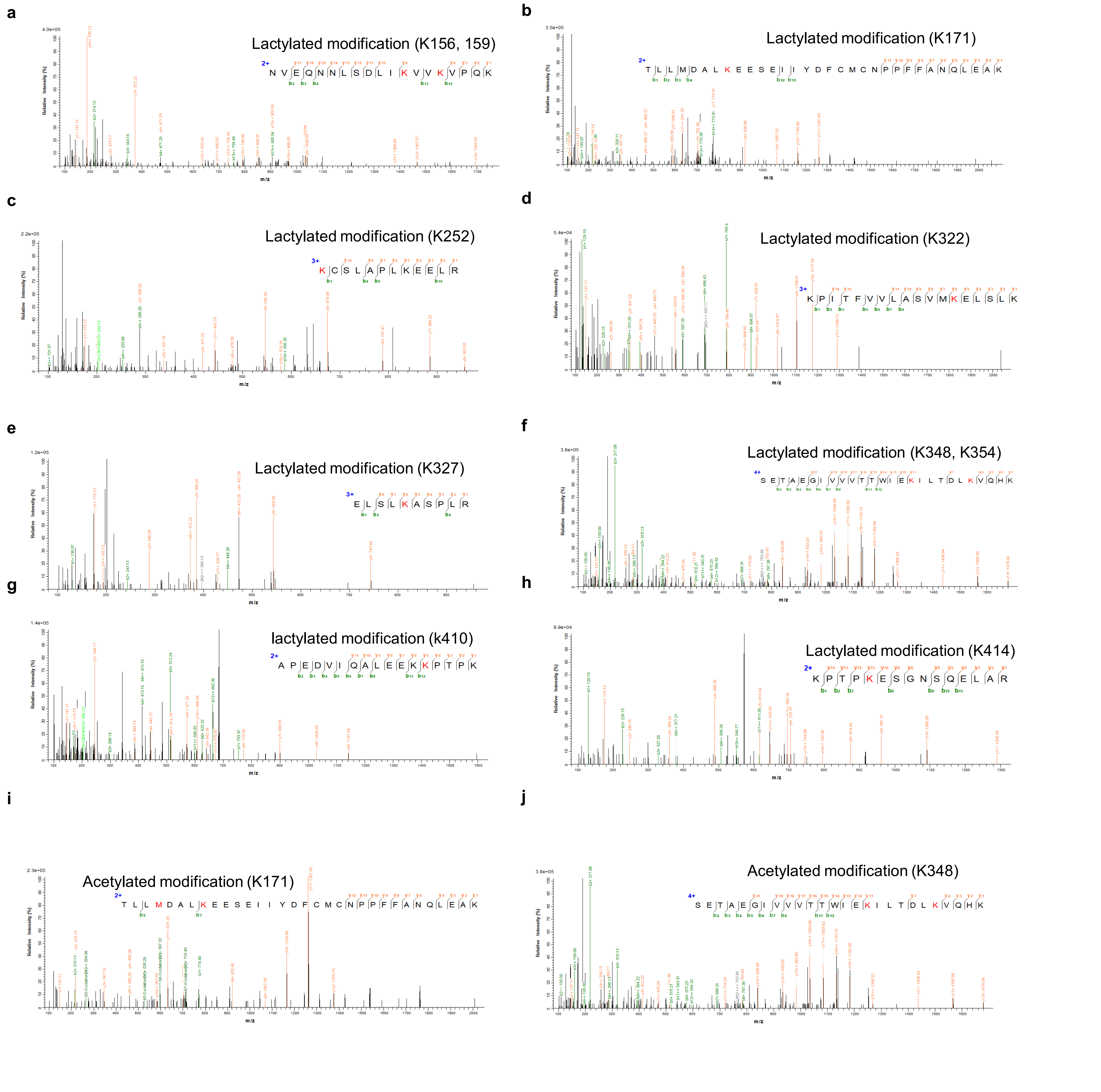

Supplementary Fig. 4. LC-MS/MS analysis showed the lactylated and acetylated sites on METTL16 protein sequence. **(a-h)** Lactylation sites include **(a)** K156 and K159, **(b)** K171, **(c)** K252, **(d)** K322, **(e)** K327, **(f)** K348 and K354, **(g)** K410, and **(h)** K414. **(i, j)** LC-MS/MS analysis showed the acetylated sites on METTL16 protein sequence. Acetylated sites include **(i)** K171, and **(j)** K348. Source data are provided as a Source data file.

Supplementary Figure 5

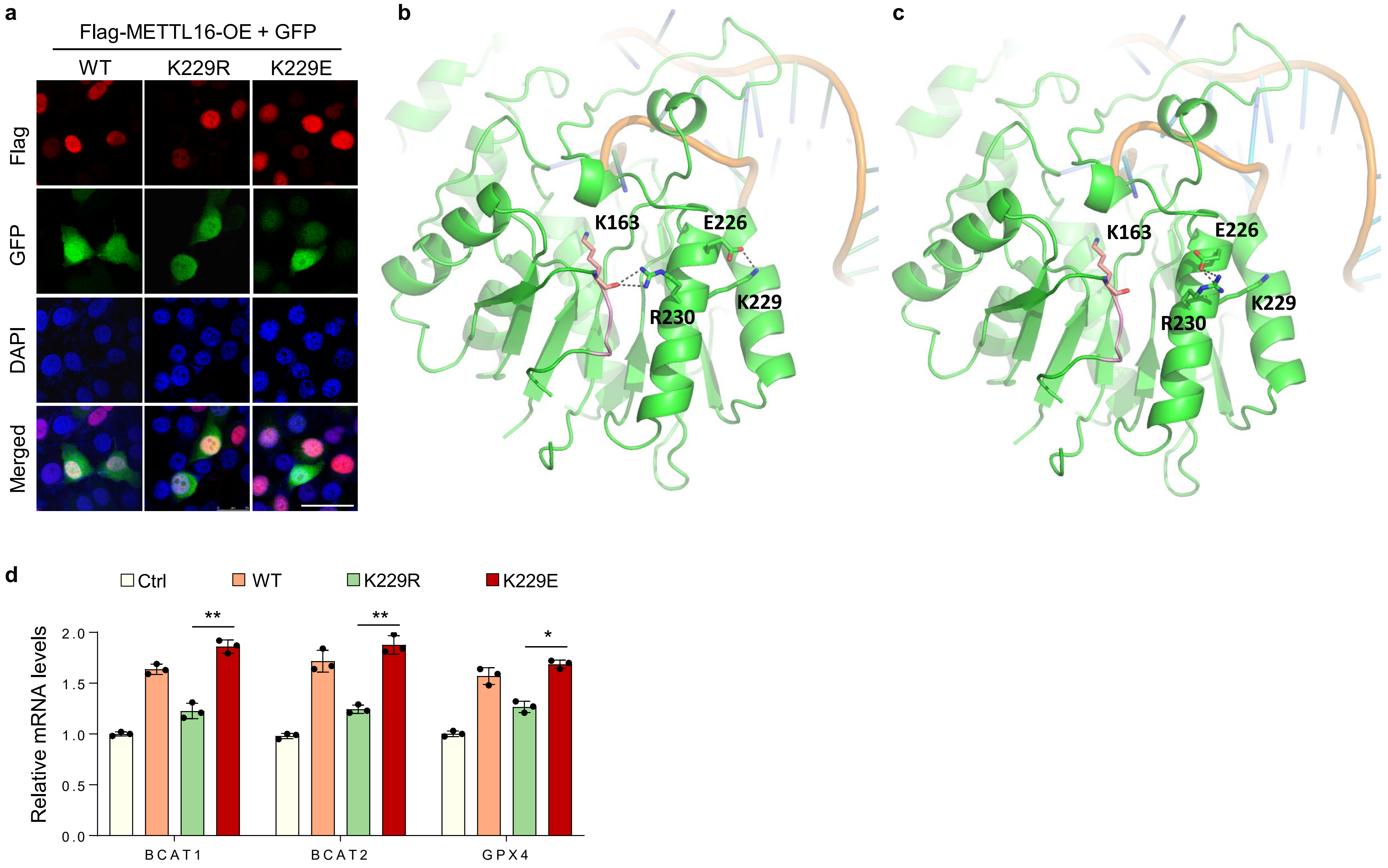

Supplementary Fig. 5. Potential impact of K229 lactylation on METTL16. (a) Immunofluorescence staining of Flag, GFP, DAPI and merged photograph showing the locations of METTL16 in HGC-27 cells transfected with Flag-METTL16-WT, -K229R, and -K229E plasmids. Scale bar, 10 µm. **(b)** Crystal structure of METTL16 complexed with MAT2A RNA hairpin (PDB 6DU5) showing that K229 formed a salt bridge with E226 and K163 from the autoinhibition loop (pink) was inserted in the SAM binding site of METTL16**. (c)** Structure model indicating that R230 could stabilize the autoinhibited state by forming hydrogen bonds with K163 or Q162 of the loop. Lactylation of K229 will abolish its salt bridge with E226, which may lead to conformational changes in sidechains of E226 and allow the formation of salt bridge with R230. This would weaken the stability of the autoinhibited state leading to elevated enzymatic activity of METTL16. The structure is shown in cartoon diagram with MEYTL16 colored in green and RNA in orange. The side chains of K163, E226, K229, and R230 are shown in sticks with nitrogen atoms in blue and oxygen in red. **(d)** qRT-PCR analysis of BCAT1, BCAT2, GPX4 expressions in Ctrl, METTL16-WT, -K229R, and -K229E cells (n=3). P (from left to right) = 0.00464, 0.00251, and 0.0146. Statistical data presented in this figure show mean values $\pm$ SD of three times of independent experiments. Statistical significance was determined by Two-tailed t test, $^{*}P < 0.05$, $^{**}P < 0.01$. Source data are provided as a Source data file

Supplementary Figure 6

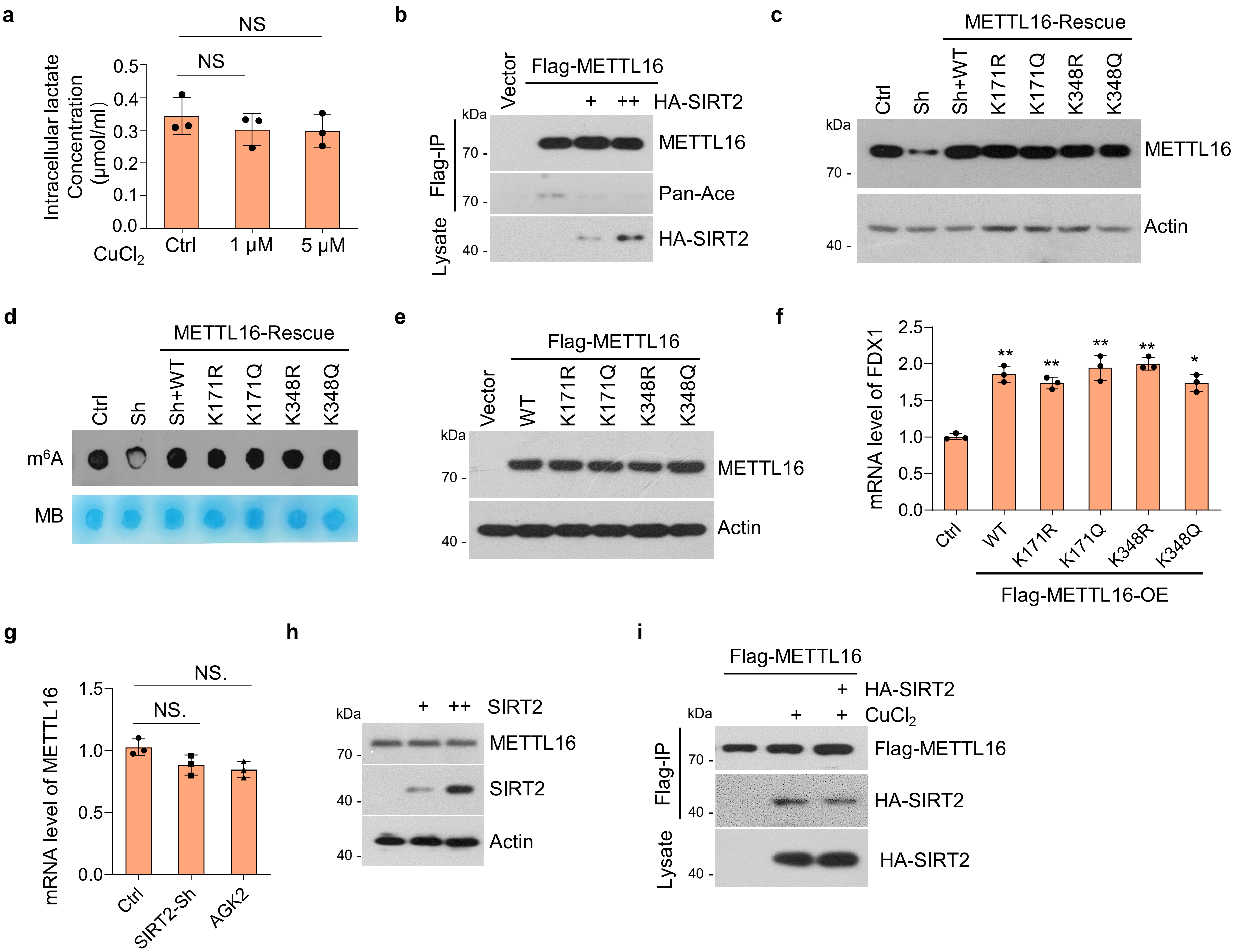

Supplementary Fig. 6. SIRT2-mediated METTL16 deacetylation has no effect on the methyltransferase activity of METTL16. (a) ELISA assay showing the intracellular lactate concentrations in Ctrl and $CuCl_2$ (1μM / 5 μM) groups using lactic acid (LA) content detection kit (n=3). P = 0.56526 and 0.85379. **(b)** Immunoprecipitation analysis of pan-Ace and METTL16 expression in HGC-27 cells transfected with Flag-METTL16 and HA-treated with different concentrations of SIRT2. **(c)** Stable cells with METTL16-Sh rescue of METTL16-WT/K171R/K171Q/K348R/K348Q. **(d)** m^6A contents in above cells treated with 5 μM $CuCl_2$ were detected by RNA dot blot. **(e)** Immunoblotting analysis of Flag-METTL16 expression in HGC-27 cells transfected with Flag-METTL16-WT, -K171R, -K171Q, -K348R, and -K348Q plasmids. **(f)** qRT-PCR analysis of *FDX1* mRNA level in HGC-27 cells transfected with indicated plasmids (n=3). WT: P = 0.0025. K171R: P = 0.0049. K171Q: P = 0.0078. K348R: P = 0.0045. K348Q: P = 0.0141. **(g)** qRT-PCR analysis of METTL16 mRNA level in Ctrl, SIRT2-Sh, or AGK2-treated cells (n=3). P = 0.2372 and 0.1250. **(h)** Western blot analysis of METTL16 expression in HGC-27 cells transfected with different amount of SIRT2 plasmid. **(i)** Flag-METTL16 and GFP-SIRT2 were co-transfected with or without HA-AARS1 or -AARS2 in 293T cells. Cell lysates were immunoprecipitated using anti-Flag antibody and western blot with anti-GFP and HA antibodies. Statistical data presented in this figure show mean values ± SD of three times of independent experiments. Statistical significance was determined by Two-tailed t test, *$P < 0.05$, **$P < 0.01$, NS = not significant. Source data are provided as a Source data file.

Supplementary Figure 7

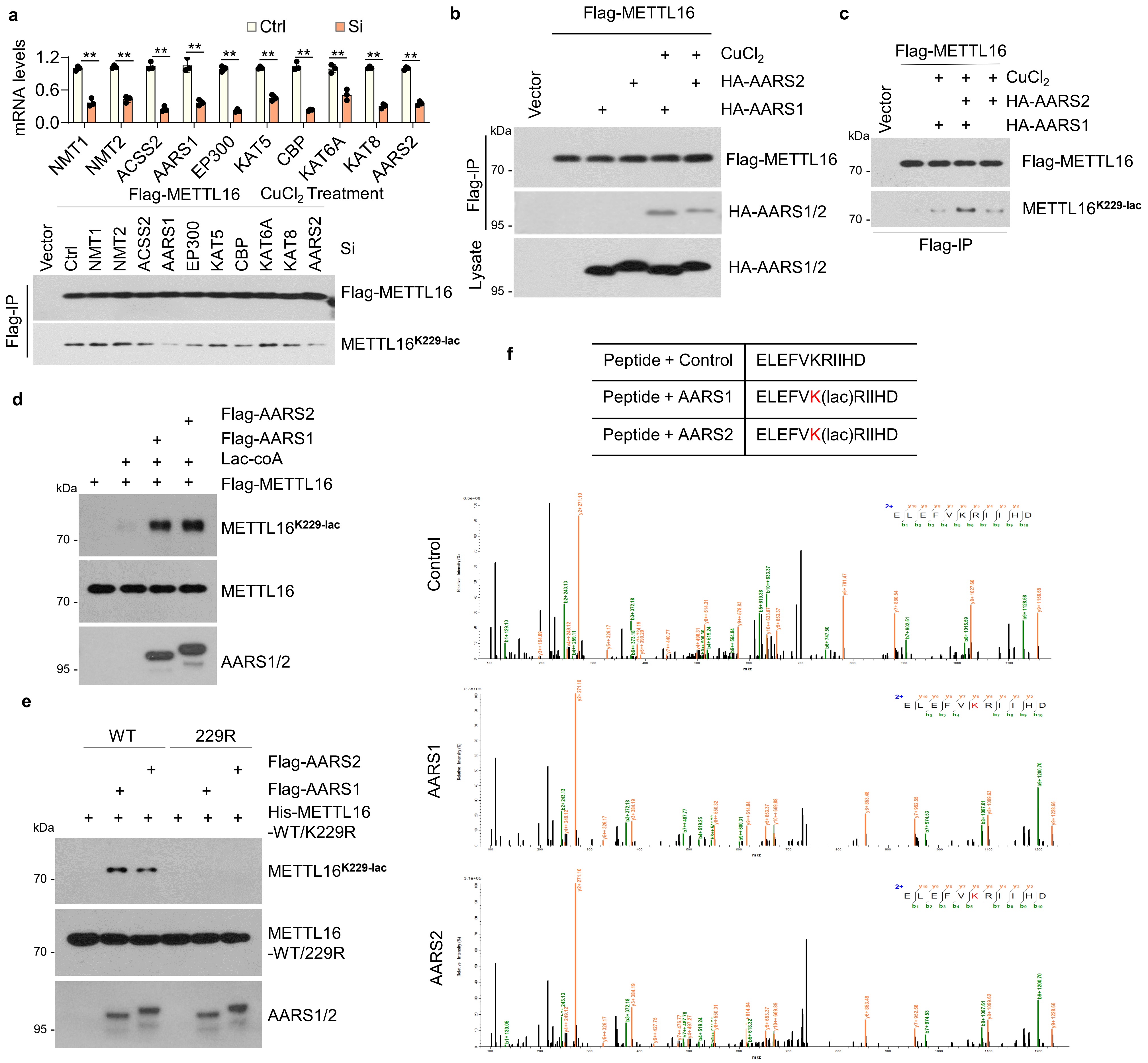

Supplementary Fig. 7. AARS1/2 directly regulate the lactylation modification of METTL16. (a) The verification of METTL16-related lactyltransferases. qRT-PCR analysis of mRNA levels of possible METTL16-related lactyltransferases in control and -siRNA cells (upper panel) (n=3). *P* (from left to right) = 0.00985, 0.00143, 0.00704, 0.00505, 0.00363, 0.00128, 0.00314, 0.010069207, 0.00071 and 0.00098. Immunoprecipitation analysis of Flag-METTL16 and METTL16$^{K229\text{-}lac}$ expression in Ctrl or transfected with indicated siRNA cells after $CuCl_2$ treatment using the anti-Flag and anti-METTL16 $^{K229\text{-}lac}$ antibodies (lower panel). **(b-c)** HGC-27 cells were transfected with HA-AARS1, HA-AARS2, or Flag-METTL16 for 15 h, and then treated with $CuCl_2$ (5 μM) for another 12 h. **(b)** The interaction of AARS1 or AARS2 with METTL16 after copper treatment. **(c)** AARS1 or AARS2 both promotes METTL16-K229 lactylation after copper treatment. (**d-f**) METTL16, AARS1, or AARS2 were precipitated from Flag -METTL16, -AARS1, or -AARS2-overexpressing 293T using FLAG-M2 beads and eluted with Flag peptide (100 μg/ml) separately. The reaction product was separated by SDS-PAGE and analyzed by western blot. (**d**) METTL16 proteins were incubated with or without AARS1/2 in the presence or absence of lactyl-CoA as indicated. **(e)** His-METTL16-WT or -229R mutants were expressed in *E. coli* and purified with His beads. His-METTL16-WT or -229R proteins were incubated with or without AARS1 or AARS2 in the presence or absence of lactyl-CoA as indicated. **(f)** A peptide of METTL16 (ELEFVK(229)RIIHD) was synthesized by Gl Biochem (Shanghai) Co., Ltd, and was incubated with AARS1, AARS2 or vector in reaction buffer (100 mM HEPES, 100 mM $MgCl_2$, 10 mM KCl, and 0.1 mM lactyl-CoA (PH = 7, Shanghai Nafu Biotechnology Co., Ltd)) at 37 ° C for 2 h. The reaction products were performed to LC-MS detection. Statistical data presented in this figure show mean values ± SD of three times of independent experiments. Statistical significance was determined by Two-tailed t test, $^*P < 0.05$, $^{**}P < 0.01$, NS = not significant. Source data are provided as a Source data file.

Supplementary Data 1. The changed genes at m^6A levels or mRNA levels after METTL16-knockdown. MeRIP-seq was performed in stable Ctrl-Sh and METTL16-Sh cell lines, and the changed genes at m^6A or mRNA levels were shown in the table. Differentially m^6A methylated peaks are identified using count based QNB test. m^6A regulation INFO: the information of m^6A modification. gene regulation INFO: the information of mRNA expression. Inf: infinite. Source data are provided as a Source data file.

Supplementary Data 2. The METTL16 binding proteins were detected by the LC-MS/MS. A total of 214 potential binding proteins of METTL16 were detected, and SIRT2 was highlighted with red color. iBAQ: intensity-based absolute quantification. Source data are provided as a Source data file.
