# Supplementary material for: Lactylation of METTL16 promotes cuproptosis via m6A-modification on FDX1 mRNA in gastric cancer
Source: Nat Commun. 2023 Oct 20;14:6523. doi: 10.1038/s41467-023-42025-8 (PMC10589265; doi:10.1038/s41467-023-42025-8)
Supplement: Supplementary file 3 — Description of Additional Supplementary Files [file 41467_2023_42025_MOESM3_ESM.pdf]

Description of Additional Supplementary Files

File Name: Supplementary Data 1
Description: **The changed genes at m^6A levels or mRNA levels after METTL16-knockdown.** MeRIP-seq was performed in stable Ctrl-Sh and METTL16-Sh cell lines, and the changed genes at m^6A or mRNA levels were shown in the table. Differentially m^6A methylated peaks are identified using count based QNB test. m^6A regulation INFO: the information of m^6A modification. gene regulation INFO: the information of mRNA expression. Inf: infinite. Source data are provided as a Source data file.

File Name: Supplementary Data 2
Description: **The METTL16 binding proteins were detected by the LC-MS/MS.** A total of 214 potential binding proteins of METTL16 were detected, and SIRT2 was highlighted with red color. iBAQ: intensity-based absolute quantification. Source data are provided as a Source data file.
