# Supplementary material for: Lactylation of METTL16 promotes cuproptosis via m6A-modification on FDX1 mRNA in gastric cancer
Source: Nat Commun. 2023 Oct 20;14:6523. doi: 10.1038/s41467-023-42025-8 (PMC10589265; doi:10.1038/s41467-023-42025-8)
Supplement: Supplementary file 6 — Reporting Summary [file 41467_2023_42025_MOESM6_ESM.pdf]

nature portfolio

Corresponding author(s): Chen Huang, Guangjian Fan

Last updated by author(s): Aug 31, 2023

Reporting Summary

Nature Portfolio wishes to improve the reproducibility of the work that we publish. This form provides structure for consistency and transparency in reporting. For further information on Nature Portfolio policies, see our Editorial Policies and the Editorial Policy Checklist.

Statistics

For all statistical analyses, confirm that the following items are present in the figure legend, table legend, main text, or Methods section.

n/a	Confirmed	
☐	☒	The exact sample size (*n*) for each experimental group/condition, given as a discrete number and unit of measurement
☐	☒	A statement on whether measurements were taken from distinct samples or whether the same sample was measured repeatedly
☐	☒	The statistical test(s) used AND whether they are one- or two-sided<br>*Only common tests should be described solely by name; describe more complex techniques in the Methods section.*
☒	☐	A description of all covariates tested
☐	☒	A description of any assumptions or corrections, such as tests of normality and adjustment for multiple comparisons
☐	☒	A full description of the statistical parameters including central tendency (e.g. means) or other basic estimates (e.g. regression coefficient) AND variation (e.g. standard deviation) or associated estimates of uncertainty (e.g. confidence intervals)
☐	☒	For null hypothesis testing, the test statistic (e.g. *F*, *t*, *r*) with confidence intervals, effect sizes, degrees of freedom and *P* value noted<br>*Give P values as exact values whenever suitable.*
☒	☐	For Bayesian analysis, information on the choice of priors and Markov chain Monte Carlo settings
☒	☐	For hierarchical and complex designs, identification of the appropriate level for tests and full reporting of outcomes
☐	☒	Estimates of effect sizes (e.g. Cohen's *d*, Pearson's *r*), indicating how they were calculated

Our web collection on statistics for biologists contains articles on many of the points above.

Software and code

Policy information about availability of computer code

Data collection	RNA quantity and quality were determined using a nanodrop (Thermo Scientific, USA). Quantitative polymerase chain reaction (PCR) amplification was performed with an Life-ABI*SimpliAmp PCR Amplifier (Life Technologies, USA). The bands resulting from gel imaging were quantified by ImageJ software (National Institutes of Health, USA). m6A sequencing was performed in LC-SCIENCE (Hangzhou, China).
Data analysis	All statistical analyzes were performed using GraphPad Prism 9.4.1 (GraphPad Software Inc., USA) and R software (version 4.2.1). For MeRIPseq, raw data was collected after the standard MeRIP process as described in Fig. 3A. QC checks were then performed. Unique mapped reads were gathered in the read mapping process using HISAT2, then peakcalling was performed by exomePeak. Then the specific motifs were collected by Motif search using HOMER. Peaks were annotated and underwent GO/KEGG analysis. RNA methylation differential analysis was then performed using exomePeak for acquiring differential peak annotations.

For manuscripts utilizing custom algorithms or software that are central to the research but not yet described in published literature, software must be made available to editors and reviewers. We strongly encourage code deposition in a community repository (e.g. GitHub). See the Nature Portfolio guidelines for submitting code & software for further information.

Data

Policy information about availability of data

All manuscripts must include a data availability statement. This statement should provide the following information, where applicable:

- Accession codes, unique identifiers, or web links for publicly available datasets
- A description of any restrictions on data availability
- For clinical datasets or third party data, please ensure that the statement adheres to our policy

The original MeRIP-seq data generated in this study have been submitted to the GEO database (accession code: GSE224890). ALL other relevant data supporting the findings of this study are available within the article and supplementary information file. Source data are provided with this paper.

Research involving human participants, their data, or biological material

Policy information about studies with human participants or human data. See also policy information about sex, gender (identity/presentation), and sexual orientation and race, ethnicity and racism.

Reporting on sex and gender	Three paired groups of GC and adjacent normal tissues were used in the present study. The first group included 48 pairs of fresh-frozen GC and adjacent normal tissue samples obtained from patients who underwent gastrectomy at Shanghai General Hospital (Shanghai, China) between October 2016 and May 2021 (female 12, male 36). The second group included 57 pairs of GC and adjacent normal tissues obtained between 2013-2014 from Shanghai General Hospital. The samples were fixed with formalin soon after surgery and embedded in paraffin to construct a tissue microarray (TMA). Three samples were lost due to producing an error, and thus we subsequently only had 54 pairs of samples (female 16, male 38). The third group included 44 plasma samples obtained from patients who underwent gastrectomy at Shanghai General Hospital (Shanghai, China) between December 2015 and October 2022 (female 12, male 32).
Reporting on race, ethnicity, or other socially relevant groupings	All the patients included are Chinese people who belong to the yellow race.
Population characteristics	The first group included 48 pairs of fresh-frozen GC and adjacent normal tissue samples obtained from patients who underwent gastrectomy at Shanghai General Hospital (the maximum age is 86, the minimum age is 36, a median age of 64.1 years). The second group included 57 pairs of GC and adjacent normal tissues obtained between 2013-2014 from Shanghai General Hospital (the maximum age is 88, the minimum age is 43, a median age of 65.9). The third group included 44 plasma samples obtained from patients who underwent gastrectomy at Shanghai General Hospital (the maximum age is 88, the minimum age is 45, a median age of 68.1).
Recruitment	All the patients recruited underwent gastrectomy with different pathological conditions at Shanghai General Hospital from 2013 to 2022. There was no self-selection bias in this study.
Ethics oversight	All samples were collected with approval from the Ethics Committee of the Shanghai General Hospital (Research Ethics Approval Code: 2022SQ123), and informed consent was obtained from all patients.

Note that full information on the approval of the study protocol must also be provided in the manuscript.

Field-specific reporting

Please select the one below that is the best fit for your research. If you are not sure, read the appropriate sections before making your selection.

☒ Life sciences ☐ Behavioural & social sciences ☐ Ecological, evolutionary & environmental sciences

For a reference copy of the document with all sections, see nature.com/documents/nr-reporting-summary-flat.pdf

Life sciences study design

All studies must disclose on these points even when the disclosure is negative.

Sample size	Sample size was chosen to ensure an adequate statistical power. All experiments were performed in technical triplicate with at least 3 independent biological replicates.
Data exclusions	In the second group of patients, 3 samples were excluded due to producing an error.
Replication	All the experiments were replicated. Three independent experiments were carried out and each experiment was performed with at least three repeats.
Randomization	All cells and the animals were randomly allocated to experimental groups.
Blinding	Blinding was not applicable in the study.

Reporting for specific materials, systems and methods

We require information from authors about some types of materials, experimental systems and methods used in many studies. Here, indicate whether each material, system or method listed is relevant to your study. If you are not sure if a list item applies to your research, read the appropriate section before selecting a response.

Materials & experimental systems

n/a	Involved in the study
☐	☒ Antibodies
☐	☒ Eukaryotic cell lines
☒	☐ Palaeontology and archaeology
☐	☒ Animals and other organisms
☒	☐ Clinical data
☒	☐ Dual use research of concern
☒	☐ Plants

Methods

n/a	Involved in the study
☒	☐ ChIP-seq
☒	☐ Flow cytometry
☒	☐ MRI-based neuroimaging

Antibodies

Antibodies used

Antibodies used in dot blot:
anti-m6A (Abclonal, A19841, Wuhan, China, 1:500)

Antibodies used in immunohistochemistry and immunofluorescent staining:
anti-METTL16 (ABclonal Technology, Wuhan, China, 1:200)
anti-FDX1 (Proteintech, USA, 1:500)
anti-SIRT2 (Servicebio, Wuhan, China, 1:1000)

Antibodies used in western blot:
lactylated METTL16-K229 (Shanghai HuiOu Biotechnology Com.LTD)
anti-β-actin (Servicebio, Wuhan, China, 1:2000)
anti-Klac (PTM Bio, Hangzhou, China, 1:1000)
anti-METTL16 (ABclonal Technology, Wuhan, China, 1:2000)
anti-FDX1 (Proteintech, USA, 1:1000)
anti-Lipoic Acid (Abcam, UK, 1:1000)
anti-DLAT (Cell Signaling Technology, USA, 1:1000)
anti-Ac (Cell Signaling Technology, USA, 1:500)
anti-Flag (Sigma-Aldrich, USA, 1:3000)
anti-GST (Cell Signaling Technology, USA, 1:1000)
anti-SIRT2 (Cell Signaling Technology, USA, 1:1000)

Secondary antibodies:
HRP-linked anti-rabbit IgG (ABclonal Technology, Wuhan, China, 1:5000);
HRP-linked anti-mouse IgG (ABclonal Technology, Wuhan, China, 1:5000).

Validation

Based on the information on the manufacturers' website, antibody was validated in wild-type and knockout samples using WB experiments, and was supported by multiple publications.

Eukaryotic cell lines

Policy information about cell lines and Sex and Gender in Research

Cell line source(s)	AGS and HGC-27 cells were purchased from the American Type Culture Collection (ATCC)
Authentication	Cell lines were not authenticated by ourselves.
Mycoplasma contamination	All the cell lines were tested negative for mycoplasma contamination.
Commonly misidentified lines (See ICLAC register)	No commonly misidentified cell lines were used in the study.

Animals and other research organisms

Policy information about studies involving animals; ARRIVE guidelines recommended for reporting animal research, and Sex and Gender in Research

Laboratory animals	Mus musculus (BALB/c) , female, age 4-6 weeks was used for the experiments.

Wild animals	No wild animals were used in the study.
Reporting on sex	We choose only female BALB/c Mus musculus for this study as sex has no influence on our study.
Field-collected samples	No field collected samples were used in the study
Ethics oversight	Animal experiments were performed according to the guidelines of the National Institutes of Health and approved by the Animal Care Committee of Shanghai General Hospital

Note that full information on the approval of the study protocol must also be provided in the manuscript.
